# Supplementary material for: Prokaryotic Diversity of the Composting Thermophilic Phase: The Case of Ground Coffee Compost
Source: Microorganisms. 2021 Jan 21;9(2):218. doi: 10.3390/microorganisms9020218 (PMC7911569; doi:10.3390/microorganisms9020218)
Supplement: Supplementary file 1 [file microorganisms-09-00218-s001.pdf]

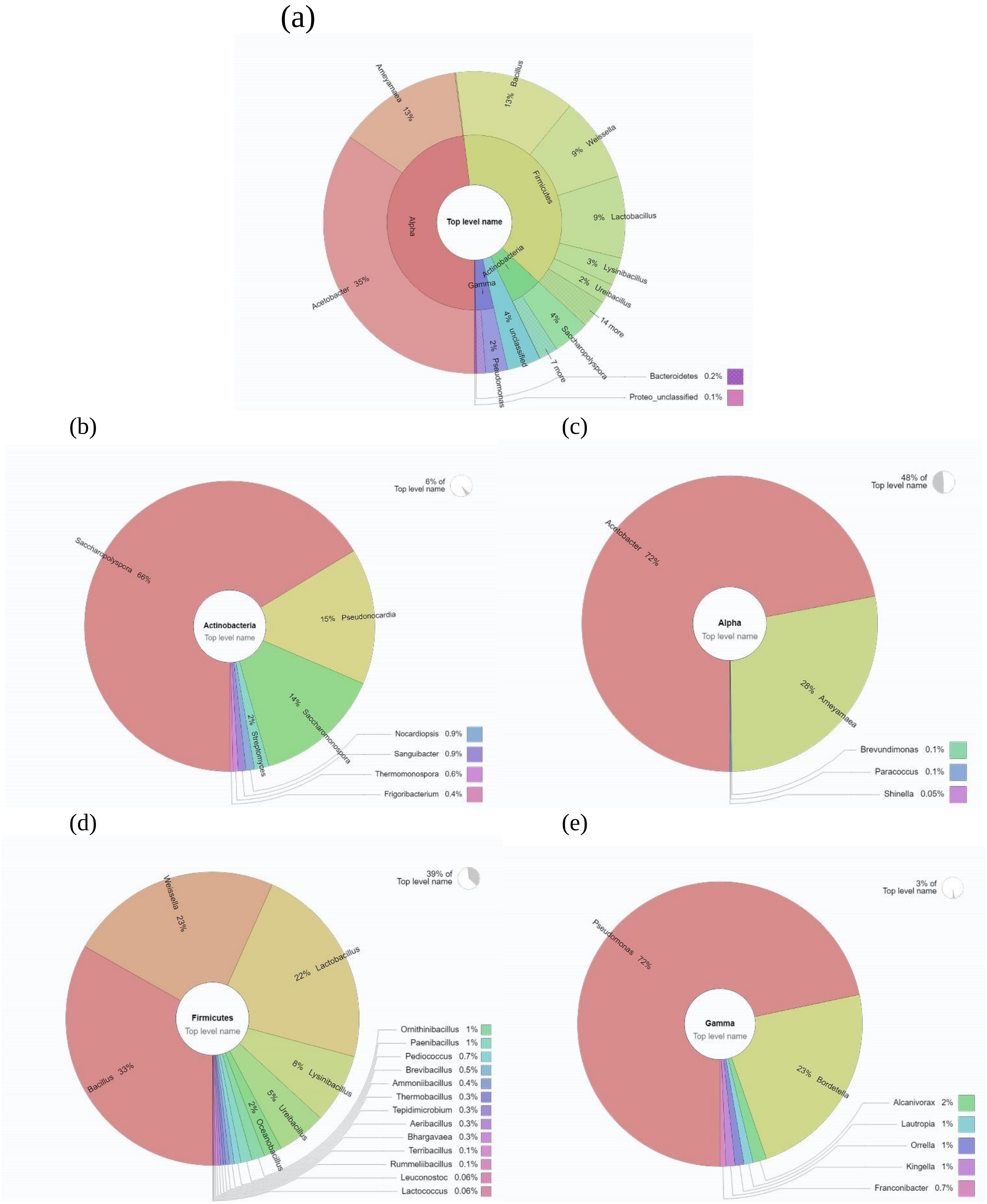

Figure S1: bacterial phylum and genera community composition. Graph generated with the krona tool. (a) Total bacterial community, (b) genera retrieved across the phylum Actinobacteria, (c) genera retrieved across the phylum of Alphaproteobacteria, (d) genera retrieved across the phylum of Firmicutes, and (e) retrieved across the phylum of Gammaproteobacteria.
